# Angiogenesis Inhibitors in Personalized Combination Regimens for the Treatment of Advanced Refractory Cancers

**DOI:** 10.3389/fmmed.2021.749283

**Published:** 2021-09-20

**Authors:** Timothy Crook, Darshana Patil, Rajnish Nagarkar, Andrew Gaya, Nicholas Plowman, Sewanti Limaye, Navin Srivastava, Dadasaheb Akolkar, Anantbhushan Ranade, Amit Bhatt, Vineet Datta, Chirantan Bose, Sachin Apurwa, Sanket Patil, Prashant Kumar, Ajay Srinivasan, Rajan Datar

**Affiliations:** ^1^ Broomfield Hospital, Chelmsford, United Kingdom; ^2^ Datar Cancer Genetics, Nasik, India; ^3^ HCG Manavata Cancer Centre, Nasik, India; ^4^ HCA Healthcare UK, London, United Kingdom; ^5^ St Bartholomew’s Hospital, London, United Kingdom; ^6^ Kokilaben Dhirubhai Ambani Hospital and Medical Research Institute, Mumbai, India; ^7^ Avinash Cancer Clinic, Pune, India; ^8^ Institute of Bioinformatics, Bangalore, India; ^9^ Manipal Academy of Higher Education, Manipal, India; ^10^ Somaiya Vidyavihar University, Mumbai, India

**Keywords:** encyclopedic tumor analysis, angiogenesis inhibitor, anti-angiogenesis, VEGF, VEGFR, PDGFR, FGFR

## Abstract

**Background:** Angiogenic factors are commonly activated in solid tumors and present a viable therapeutic target. However, anticancer treatment with angiogenesis inhibitors (AGI) is limited to a few cancers, mostly as monotherapy and not selected based on molecular indications. We aimed to determine whether patient-specific combination regimens with AGI and other anticancer agents when selected based on multi-analyte tumor interrogation (ETA: Encyclopedic Tumor Analysis) can expand the scope of AGIs in advanced refractory solid organ cancers with improved treatment responses.

**Methods:** We evaluated treatment outcomes in 60 patients with advanced, refractory solid organ cancers who received ETA-guided combination regimens of AGI with other targeted, endocrine or cytotoxic agents. Radiological evaluation of treatment response was followed by determination of Objective Response Rate (ORR), Disease Control Rate (DCR), Progression Free Survival (PFS) and Overall Survival (OS).

**Results:** Among the 60 patients, Partial Response (PR) was observed in 28 cases (46.7%), Stable Disease (SD) was observed in 29 cases (48.3%) and Disease Progression (PD, within 60 days) was observed in 3 cases (5.0%). The ORR was 46.7% and DCR was 95.0%. At the most recent follow-up the median PFS (mPFS) was 5.0 months and median OS (mOS) was 8.9 months. There were no Grade 4 therapy related adverse events or treatment related deaths.

**Conclusion:** ETA-guided patient-specific combination regimens with AGI and other anti-neoplastic agents, can yield improved outcomes over AGI monotherapy. Trial Registration: Details of all trials are available at WHO-ICTRP: https://apps.who.int/trialsearch/. RESILIENT ID CTRI/2018/02/011,808. LIQUID IMPACT ID CTRI/2019/02/017,548.

## Introduction

Tumor angiogenesis is one of the hallmarks of cancer (Cook and Figg, 2010; El-Kenawi and El-Remessy, 2013). Angiogenic factors such as VEGF (Hicklin and Ellis, 2005), PDGF (Gialeli et al., 2014; Farooqi and Siddik, 2015), FGF (Korc and Friesel, 2009), c-KIT (Foster et al., 2018), RET (Jhiang, 2000), TIE (Partanen and Dumont, 1999) and their receptors (tyrosine kinases) such as VEGFR, PDGFR, FGFR mediate inter- and intra-cellular signalling cascades that activate cellular pathways culminating in the formation and branching of blood vessels which promotes rapid tumor growth. These angiogenesis pathways are known to cross-talk (Lai et al., 2018) with other key tumor signalling pathways such as the PI3K/Akt/mTOR (Conciatori et al., 2018), EGFR/ERBB2 (van Cruijsen, Giaccone, and Hoekman, 2005) and Raf-MEK-ERK-MAPK (Song and Finley, 2018) in a complex web of redundant and escape mechanisms which are favourable for tumor growth and proliferation; blockade of these signalling pathways is a viable strategy to control tumor growth and spread (Pietras et al., 2003; Press and Lenz, 2007; Shibuya, 2011; Sitohy, Nagy, and Dvorak, 2012; Dieci et al., 2013; Heldin, 2013; Stankov, Popovic, and Mikov, 2014; Abbaspour Babaei et al., 2016; Navid et al., 2020; Subbiah and Cote, 2020). Several anti-angiogenic agents (angiogenesis inhibitors, AGIs) including monoclonal antibodies (mABs) such as Bevacizumab (Shih and Lindley, 2006) (anti-VEGF) or Ramucirumab (Fala, 2015) (anti-VEGFR2) and small molecule Tyrosine Kinase Inhibitors (TKIs) such as Axitinib (Rini et al., 2011), Pazopanib (Sternberg et al., 2010; van der Graaf et al., 2012), Regorafenib (Demetri et al., 2013; Grothey et al., 2013; Bruix et al., 2017), Sorafenib (Wilhelm et al., 2004; Llovet et al., 2008; Brose et al., 2014) and Sunitinib (G D Demetri et al., 2005; Ravaud et al., 2016; Raymond et al., 2011) have been developed and approved for use in various cancers; though Imatinib (Dagher et al., 2002) is not a direct inhibitor of angiokinases, its activity has been linked to downstream suppression of VEGF expression and thus angiogenesis. Unlike the mABs which are specific to a target, anti-angiogenic TKIs are known to have intracellular activity and downstream signalling a broad range of targets such as VEGFR, PDGFR, FGFR, TIE, c-KIT, RET and RAF. An overview of AGIs used in several solid organ cancers is presented in Table 1 along with molecular targets and indicated usage.

**TABLE 1:** Details of Angiogenesis Inhibitors (AGIs) administered in the present study, their targets used in epithelial and solid organ cancers. Apart from Bevacizumab which is a monoclonal antibody (mAb) specific for VEGF, all other AGIs are small molecular tyrosine kinase inhibitors (TKIs) which can inhibit multiple targets. Combinations of Bevacizumab are frequently approved in several cancers. Apart from Axitinib, other TKIs are largely used as monotherapy. None of the AGIs are selected on the basis of tumor profiling for these targets.

Drug	Target(s)	Use in cancers
Bevacizumab	VEGF	GBM; CRC^1^; NSCLC^2^; Cervix^3^; Ovary^4^; RCC^5^; HCC^6^
Axitinib	VEGFR, *PDGFR*, *c-KIT*	RCC^7^
Imatinib	BCR-ABL, *PDGFR*, *c-KIT*	GIST
Pazopanib	VEGFR, PDGFR, c-KIT, FGFR	RCC, Sarcomas
Regorafenib	VEGFR, PDGFR, c-KIT, RET	CRC, GIST, HCC
Sorafenib	VEGFR, PDGFR	RCC, HCC, Thyroid
Sunitinib	VEGFR, PDGFR, c-KIT, RET	GIST, Pancreatic NET, RCC

GBM: Glioblastoma; CRC: Colorectal Cancer, NSCLC: Non-Small Cell Lung Cancer; RCC: Renal Cell Carcinoma; HCC: Hepatocellular Carcinoma; GIST: Gastrointestinal Stromal Tumor; NET: Neuroendocrine Tumor. ^1^with FOLFOX/FOLFIRI/FOLFIRINOX; ^2^with Carboplatin and Paclitaxel; ^3^with Paclitaxel and Cisplatin/Topotecan; ^4^with Paclitaxel and Carboplatin; ^5^with IFN-α; ^6^with Atezolizumab; ^7^as monotherapy as well as with Pembrolizumab/Avelumab.

It has been shown that blockade of VEGF/VEGFR signalling axis by monotherapies often leads to transient responses followed by eventual resistance and progression (Zhao and Adjei, 2015; Haibe et al., 2020). Similarly, combination strategies that achieve tandem blockade of multiple signalling pathways have been proposed as a viable anticancer treatment strategy (Gotink and Verheul, 2010; Qin et al., 2019; Yi et al., 2019; Deng et al., 2020). Since the role of the Notch signaling pathway in Angiogenesis is well recognized (Akil et al., 2021; Majumder et al., 2021), targeting angiogenesis via inhibition of the Notch pathway is also considered as a viable anti-cancer treatment approach. However, apart from Bevacizumab (Axitinib to a limited extent due to its use in combinations with Immune Checkpoint Inhibitors) most AGIs have been used as monotherapy and as such they offer limited therapeutic benefit. Secondly, though the molecular targets of AGIs are known, the selection of AGIs is not on the basis of tumor molecular profiling; currently there is no reliable molecular biomarker for selection of these agents. It is pertinent to add that prior efforts at informing use of targeted agents based on limited (single gene) tumor molecular profiling have yielded discouraging outcomes. Owing to these challenges, the aim of precision oncology to offer personalized (combination) regimens remains unachieved.

We have previously reported (Nagarkar et al., 2019; Ranade et al., 2019) the clinical utility of multi-analyte tumor profiling (ETA: Encyclopedic Tumor Analysis) to identify latent actionable vulnerabilities in advanced refractory cancers and guide selection of safe and efficacious patient-specific combination regimens. We hypothesized that ETA can be used to inform personalized combination regimens of AGIs with other anti-neoplastic agents, which can yield superior or improved clinical outcomes in patients with advanced refractory solid organ cancers.

## Methods

### Study Design and Patients

This manuscript reports outcomes in a total of 60 adult patients who received anticancer treatment regimen informed by multi-analyte tumor profiling (ETA guided therapy). Among this cohort, 55 patients with advanced solid organ cancers, received treatments as part of either of the two ongoing prospective interventional Phase II/III trials; 38 patients in RESILIENT trial (WHO ICTRP ID CTRI/2018/02/011,808) and 17 patients in LIQUID IMPACT trial (WHO ICTRP ID CTRI/2019/02/017548), into which they were enrolled between January 2018 and October 2019. All patients were previously counselled regarding study objectives, potential benefits and potential risks and provided signed written informed consent for participation in the trial and for publication of de-identified data. Both trials were approved by the ethics committees (EC) of the sponsor as well as clinical trial sites and conducted in accordance with ethical guidelines and the Declaration of Helsinki. The primary outcome data for the RESILIENT Trial has been published (Ranade et al., 2019) while that of the LIQUID IMPACT trial is under submission. In addition, we also report outcomes in 5 adult patients with advanced solid organ cancers who underwent ETA as a commercial service between December 2016 and June 2018 to inform precision systemic therapy options. All patients consented for analysis and publication of de-identified data. Demographic details of the Study Cohort are provided in Table 2 and Supplementary Table S1.

**TABLE 2:** Study Cohort. The present study reports outcomes in a curated cohort of 60 adult patients with advanced refractory solid organ cancers.

Parameter	Value
Gender	
Female	30
Male	30
Age	
Median (Range)	49 (22–71)
Cancer Type	
Breast (IDC)	11
Cervix (NET)	1
Colorectum (AD)	5
Duodenum (NET)	1
Esophagus (SCC)	2
Head and Neck (SCC)	13
Kidney (RCC)	3
Liver (HCC)	2
Lung (AD)	1
Lung (SCC)	1
Melanoma	1
Occult (NET)	2
Ovary (AD)	7
Pancreas (AD)	3
Pilomatrixoma	1
Sarcoma	1
Stomach (AD)	4
Testes (NSCGT)	1

IDC: Invasive/Infiltrating Ductal Carcinoma; SCC: Squamous Cell Carcinoma; AD: Adenocarcinoma; NET: Neuroendocrine Tumor; RCC: Renal Cell Carcinoma; HCC: Hepatocellular Carcinoma; PMX: Pilomatrixoma; NSGCT: Non-Seminomatous Germ Cell Tumor.

### Samples and Analysis

All patients in the RESILIENT Trial (n = 38) as well as the non-trial patients (n = 5) provided freshly biopsied tissue samples along with peripheral blood samples obtained by venous draw. All patients in the LIQUID IMPACT Trial (n = 17) provided only peripheral blood samples. The collection of blood samples has been described previously (Nagarkar et al., 2019; Ranade et al., 2019). ETA and generation of patient specific therapy recommendations (TR) have been described previously (Nagarkar et al., 2019; Ranade et al., 2019). Briefly, ETA included 1) molecular profiling of tumor DNA, circulating tumor DNA (ctDNA), tumor RNA and exosomal RNA by Next Generation Sequencing (NGS), 2) immunocytochemistry (ICC) of Circulating Tumor Associated Cells (CTACs), and, 3) *in vitro* Chemoresponse Profiling (CRP) of viable tumor tissue derived cells (TDCs) or CTACs. The complete details of investigations under ETA are provided in Supplementary Methods. Supplementary Table S2 indicates the various actionable molecular indications in the Study Cohort, as informed by ETA.

### Treatments

Among the 60 patients who received AGI-based regimen, 7 received additional targeted/endocrine agents, 43 received additional cytotoxic agents and 10 received additional targeted/endocrine and cytotoxic agents. A break-up of ETA-guided treatment regimen including AGIs, other Targeted and Endocrine agents and cytotoxic anticancer agents is provided in Supplementary Table S2. The selection of patient-specific combination regimens, the drug dosages and dose escalation schedules were based on the treating clinician’s interpretation of ETA findings and guided by 1) institutional guidelines and protocols, 2) clinical assessment of patient’s health, and 3) patient-wise list of expected treatment-related Adverse Events (AE), generated from known AE profile of single agents (from drug labels) and their combinations (drug labels and clinical trial data). Treatments were administered until disease progression or dose limiting toxicity.

### Response Evaluation

Treatment response was assessed radiologically based on a baseline and follow-up (PET-)CT scans as per RECIST 1.1 criteria (Eisenhauer et al., 2009) to determine Treatment Response as Partial Response (PR), Stable Disease (SD) or Progressive Disease (PD). Treatment response was evaluated to derive Objective Response Rate (ORR), Disease Control Rate (DCR), Progression Free Survival (PFS) and Overall Survival (OS). Trial patients underwent follow-up scans every 6–8 weeks or as advised by the treating clinician. Non-trial patients underwent follow-up scans at intervals advised by the treating clinicians.

### Follow-Up

Trial patients were followed up until study termination or patient exclusion (death/loss to follow-up/withdrawal of consent) to determine Progression Free Survival (PFS) and Overall Survival (OS). Post completion of (or exclusion from) the respective study, patients were followed-up for OS.

### Safety and Adverse Events

Treatment related AEs were prospectively recorded for trial patients and retrospectively for the non-trial patients from clinical records provided by the treating clinician. All AEs were graded according to NCI-CTCAE v5 (NCI, NIH, DHHS, 2017) and reported. For all patients, AEs were managed by standard procedures according to institutional protocols.

## Results

### Molecular Profile

**FIGURE 1:**
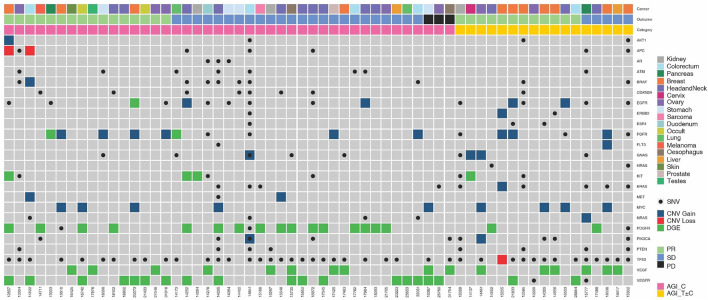
Molecular Landscape of Angiogenesis Factors and their Receptors in the Study Cohort. Molecular features associated with angiogenesis factor and receptor genes are depicted. Five-digit numbers at the bottom of each column indicate individual patients in the study cohort. Cancer types (topmost row) and gender (second row from top) are colour coded. SNV: Single Nucleotide Variation; CNV: Copy Number Variation; PR: Partial Response; SD: Stable Disease; PD: Disease Progression.

The molecular landscape of angiogenesis associated genes in the study cohort is depicted in Figure 1. The most common indications were observed in PDGFR (n = 21), VEGFR (n = 19), and VEGF (n = 18), followed by c-KIT (n = 8) and FGFR (n = 5). Gene overexpression as determined by tumor RNA analysis was the most common variation (n = 46), followed by protein detection by ICC (n = 16). In 8 cases, mutations were observed and in 6 cases, gain of gene copy number was observed. Indications in more than 1 gene and/or more than one type of variation per gene were observed in 9 patients. Patient-wise actionable molecular features which were relevant for selection of AGIs as well as other Targeted/Endocrine anti-cancer agents are provided in Supplementary Table S2.

### Treatments

Among the 60 patients, the multi-drug regimens included an AGI with ≥1 cytotoxic agent in 43 patients and an AGI with ≥1 targeted/endocrine agents in 7 patients; 10 patients received an AGI with other targeted/endocrine agents as well as ≥1 cytotoxic agent(s). Only USFDA approved anticancer drugs were used in patient specific combination regimens. Selection of cytotoxic, targeted, and endocrine agents were agnostic to the respective labelled indications. Patient-wise treatment regimens including choice of AGIs, other Targeted and Endocrine agents and cytotoxic anticancer agents as well as the dosages are provided in Supplementary Table S2.

### Treatment Response

Among the 60 patients who received ETA-guided combination regimens, there were no Complete Responses (CR), while 28 patients (46.7%) showed Partial Response (PR), 29 patients (48.3%) showed SD (≥60 days) and 3 (5.0%) patients showed PD within 60 days of therapy. The Objective Response Rate (ORR) in this sub-cohort was 46.7% and Disease Control Rate (DCR) was 95.0%. Patient out comes are summarized in Table 3 and Supplementary Table S3. Among the 43 patients who received combination of AGI and cytotoxic agents (AGI_C), PR was observed in 16 patients (37.2%). Among the remaining 17 patients where the combination regimen included an additional targeted or endocrine agent (AGI_T ± C) PR was observed in 12 patients (70.6%). None of the patients who received multiple targeted agents (AGI_T ± C) reported PD within 60 days; all 3 patients who reported PD within 60 days were from the AGI_C subgroup.

**TABLE 3:** Outcomes of ETA-guided Combination Regimens of AGI and other Targeted, Endocrine and Cytotoxic agents.

Parameter	Overall	AGI_C	AGI_T ± C
Patients	60	43	17
Outcome			
PR	28 (46.7%)	16 (37.2%)	12 (70.6%)
SD	29 (48.3%)	24 (55.8%)	5 (29.4%)
PD	3 (5.0%)	3 (7.0%)	-
Response Rates			
ORR	46.7%	37.2%	70.6%
DCR	95.0%	93.0%	100.0%
Survival*			
mPFS	5.0 (4.1–5.8)	4.4 (3.4–5.3)	6.7 (4.8–8.5)
mOS	8.9 (7.3–10.6)	8.8 (6.7–10.9)	10.0 (7.2–12.9)

PR: Partial Response; SD: Stable Disease; PD: Progressive Disease; ORR: Objective Response Rate; DCR: Disease Control Rate; mPFS: Median Progression Free Survival (months); mOS median Overall Survival (months). *PFS and OS data as well as the 95% CI are reported as on the most recent date of follow-up.

### Progression Free Survival and Overall Survival

**FIGURE 2:**
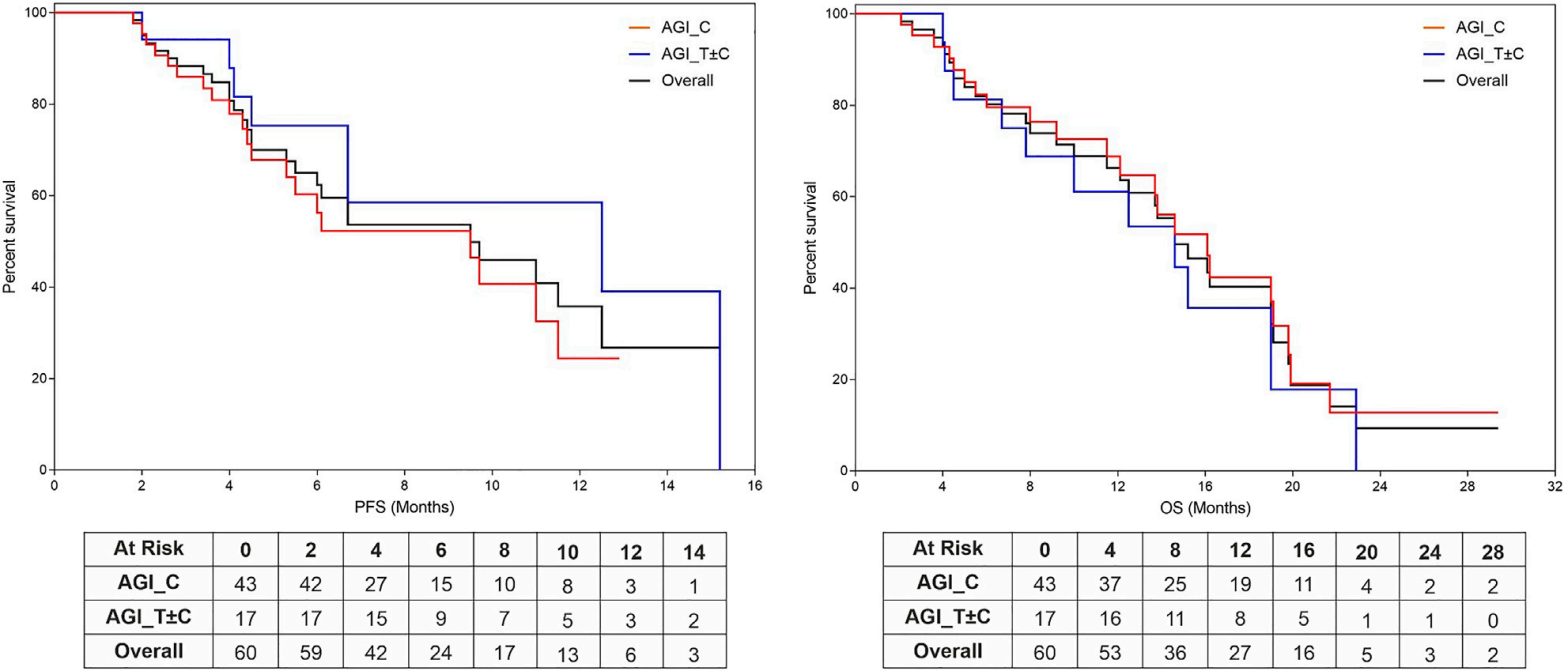
Kaplan Meier Plots of Progression Free Survival and Overall Survival. Progression Free Survival (PFS, **(A)**) and Overall Survival (OS, **(B)**) were evaluated based on regimen subtypes: AGI in combination with Cytotoxic Agents (AGI_C) or other targeted agents irrespective of cytotoxic agents (ETA_T ± C).

Among the 60 patients, median Progression Free Survival and Median Overall Survival (mPFS and mOS, respectively) were 5.0 months (95% CI: 4.1–5.8 months) and 8.9 months (95% CI: 7.3–10.6 months) respectively. Among the 43 patients in the AGI_C subgroup, mPFS and mOS were 4.4 and 8.8 months respectively, whereas in the AGI_T ± C subgroup, these values were incrementally higher 6.7 and 10.0 months, respectively (the cohort sizes do not permit calculation of statistical significance). Kaplan Meier Plots of PFS and OS are presented in Figure 2.

**FIGURE 3:**
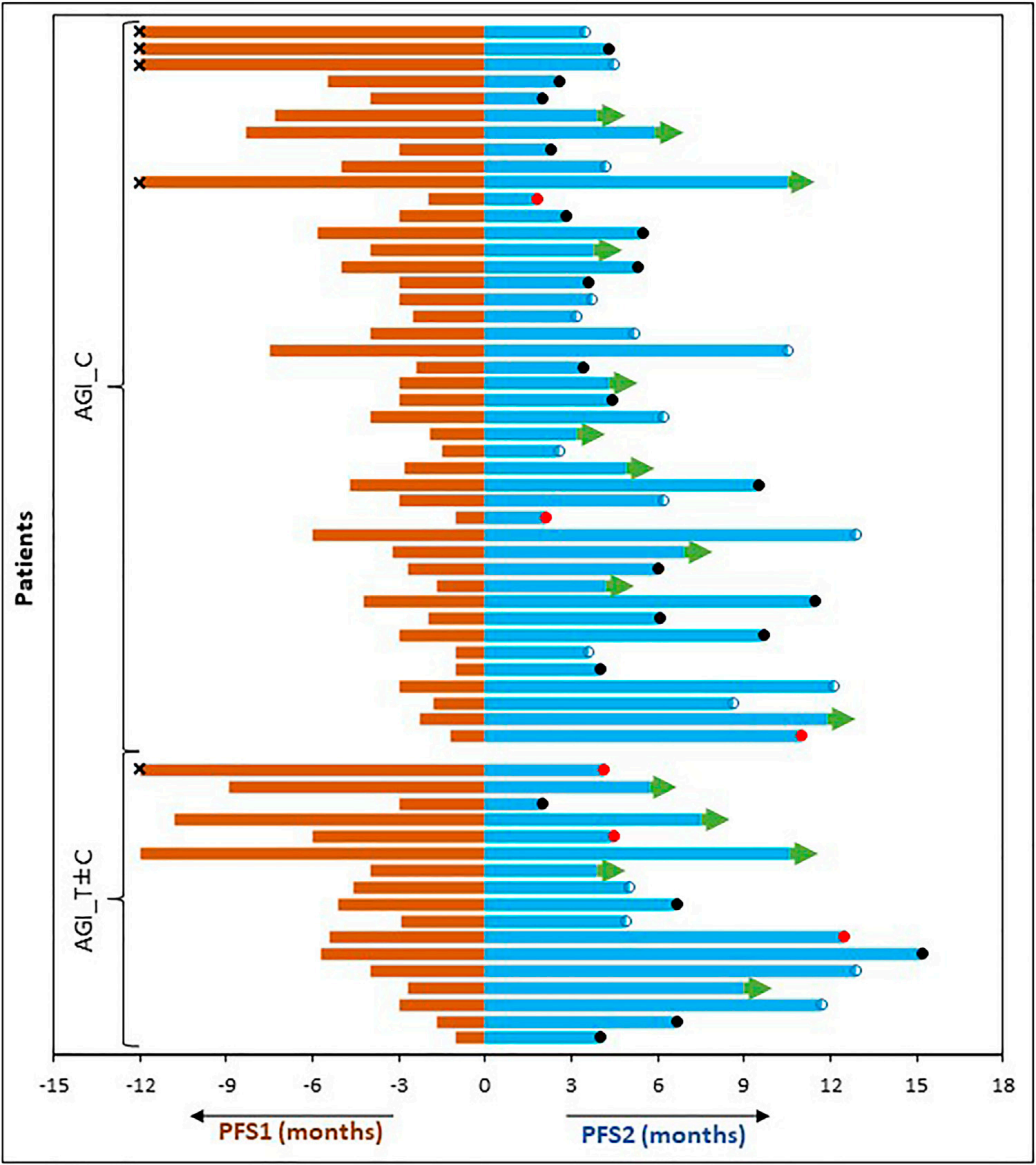
Comparison of Progression Free Survival on Last Failed Line of Systemic Treatment (PFS1) and on the Present Regimen (PFS2). PFS of each patient (months) on the last line of treatment **(left)** is compared with the PFS observed on ETA guided AGI combination therapy regimen **(right)**. censored. ●: demise; ●: progression; →: ongoing PFS; **×**: PFS1 was >12 months.

We benchmarked the PFS of each patient on ETA-guided combination regimen (PFS2) against PFS on patient’s last failed line of therapy (PFS1) (Figure 3). With a median PFS1 of 3.1 (95%CI: 1.1–5.1 months) months, the overall PFS2:PFS1 ratio was 1.6, indicating therapeutic benefit leading to significant increase in PFS. The PFS2:PFS1 ratios were ≥2.50 in 16 patients (26.7%), 1.31–1.49 in 19 patients (31.7%) and 0.81–1.29 in 13 patients (21.7%). Among the 12 patients with PFS2:PFS1 ratio of ≤0.8, there were 6 patients with recurrent disease (>12 months gap). Patient outcomes are summarized in Table 3 and Supplementary Table S3.

### Treatment Related Adverse Events

Grade I/II Treatment-Related Adverse Events (AE) were observed in all 60 patients (Table 4). Treatment related Grade III AEs were observed in 45 patients. Grade IV treatment related AEs were not reported, nor were any treatment related deaths. The most common Grade I/II AEs included Fatigue, Anorexia, Mucositis, Nausea, which also were reported as Grade III AEs albeit at lower frequencies. There were no significant differences between profiles of AEs depending on the nature of the treatment regimen (i.e., AGI_C v/s AGI_T ± C). Patient-wise AEs are provided in Supplementary Table S4.

**TABLE 4:** Profile of Known and Likely Treatment Related Adverse Events. There were no recorded Grade IV Treatment Related AEs nor any Treatment Related Deaths. Patient-wise AEs reported as well as AEs in treatment subgroups (AGI_C, AGI_T ± C) are provided in Supplementary Table S4.

Adverse event	Grade I/II		Grade III	
Anemia	9	15.0%	4	6.7%
Anorexia	41	68.3%	9	15.0%
Constipation	9	15.0%	2	3.3%
Diarrhoea	13	21.7%	2	3.3%
Edema	11	18.3%	3	5.0%
Fatigue	54	90.0%	28	46.7%
Hyper-/Hypotension	5	8.3%	3	5.0%
Mucositis Oral	27	45.0%	9	15.0%
Myalgia	14	23.3%	4	6.7%
Nausea	21	35.0%	4	6.7%
Neutropenia	17	28.3%	5	8.3%
Peripheral Neuropathy	6	10.0%	2	3.3%
Rash/Itch	10	16.7%	2	3.3%
Pyrexia	15	25.0%	2	3.3%
Thrombocytopenia	12	20.0%	10	16.7%
Vomiting	11	18.3%	1	1.7%

## Discussion

The findings of the present study show that co-administration of AGI and other anticancer agents are well tolerated and achieve significant response even in a heavily pre-treated cohort with advanced solid organ cancers. It is generally accepted that the probability of success of anticancer treatments decrease with successive lines of treatment. However, our study shows a significant increase in median PFS as compared to the patient’s last failed line of treatment. Since several patients are continuing to receive treatments at the time of submission, we anticipate further improvements to the treatment benefit metrics reported in this manuscript.

Presently, the selection of AGI for use in anticancer treatment regimens is not informed by molecular genetic indication. This is the case for most, if not all, targeted anticancer agents such as tyrosine kinase inhibitors (TKI) as well as mABs. Though the paradigm of personalized label agnostic treatment selection based on tumor molecular profiling has been explored for several targeted anticancer agents, this appears to be less so in case of AGIs where there are fewer studies on organ agnostic treatment selection. Among the NCI-MATCH (NCI-MATCH, 2020)basket trials, there are 3 trials for FGFR aberrations and 1 trial on cKIT aberrations for selection of AGI; interim results for one of these studies indicated absence of significant treatment benefit with 9% ORR (Chae et al., 2020). Most AGIs are administered as monotherapy which yield modest treatment benefits. Prior studies have established the benefits of combination regimens of various TKIs in comparison to monotherapy with the same agents. In several cancers, targeted agents are administered with other targeted or endocrine or cytotoxic agents; Axitinib and Bevacizumab appear to be researched more extensively in comparison to other AGIs reported in this manuscript. Several combinations of Axitinib and Bevacizumab with other targeted agents, checkpoint inhibitors or cytotoxic agents are presently approved for treatment of various cancers since they offer varying levels of improvement over existing monotherapy options. In a recent study (Donato et al., 2020) it was observed that VEGF blockade, though effective in suppressing the primary tumour, could present hypoxic conditions conducive to release and survival of CTC clusters with metastatic potential. It follows that a combination regimen that targets angiogenesis as well as other activated pathways or cellular mechanisms (such as e.g., DNA replication, DNA synthesis, or mitosis) in tandem could avoid such potential pitfalls by acting on CTCs or clusters released from the tumor which are no longer susceptible to inhibition of angiogenesis.

The present study was designed to select AGIs based on molecular indications for use in patient specific combination regimens with other anticancer drugs which were selected based on *de novo* functional and molecular profiling of the tumors. The scope of AGIs in the present study was limited by the availability of approved drugs at the trial location and included Axitinib, Bevacizumab, Pazopanib, Regorafenib, Sorafenib and Sunitinib; Imatinib, though not a classical AGI is known to inhibit angiogenesis as a downstream function and was also considered. Except for Bevacizumab, a mAb specific for VEGF, all other anticancer agents are small molecule TKIs with varying activities against a repertoire of angiogenesis-related factors including VEGFR, PDGFR, FGFR, c-KIT, RET, and RET families. Accordingly, ETA evaluated gene alterations including single nucleotide variations (SNV) and copy number alterations (CNA, specifically gain of copy number) as well as overexpression of all genes where the polypeptide product can be targeted by AGIs. While SNV and CNA were determined by NGS, gene expression was evaluated by NGS of tumor tissue RNA or exosomal RNA, as well as by ICC of peripheral blood C-TACs. The study cohort included 18 patients who were unable to undergo a biopsy for tumor profiling–in these patients, peripheral blood tumor analytes including circulating tumor DNA (ctDNA), exosomal RNA and C-TACs were evaluated.

The molecular profiles of angiogenesis related genes in each patient are illustrated in Figure 1. In 21 patients, molecular features associated with multiple angiogenesis related genes was observed while in 3 patients, multiple alterations in same gene (copy number variation as well as point mutation) were observed. This aided the selection of AGIs that can efficiently target multiple vulnerabilities. In 17 patients, ETA also identified other tumor features such as (but not limited to) activation of mTOR or hormone receptor signalling pathways. In these patients, the corresponding targeted agent or endocrine antagonist was evaluated for incorporation in the treatment regimen.

The study findings suggest that ETA based comprehensive multi-analyte cancer profiling provides relevant molecular and functional evidence which in turn can inform selection of patient-specific combination regimens of AGIs with other anticancer agents. The benefits of such combination regimens may exceed those obtained with arbitrary selection of monotherapy AGIs or their combination regimens, respectively. While the merits of combination treatment strategies have been expounded previously (Liu, Nikanjam, and Kurzrock, 2016; Nikanjam, Liu, and Kurzrock, 2016; Nikanjam et al., 2017), the safety of such regimens are an important consideration. Meta-analyses of clinical trials have observed that *de novo* combinations of targeted and cytotoxic agents can be safely administered to patients without any increased risks of toxicity. The present study comprised exclusively of heavily pre-treated cases of advanced cancers with an inherently higher risk of AEs due to cumulative toxicities from prior treatments; ETA guided combination regimens of AGI and other anticancer agents were generally well tolerated with manageable profile of treatment related AEs. Though the present study did not include any therapy naïve patients, we speculate that ETA-guided approach can be beneficial as first line for advanced metastatic cases or in the neoadjuvant setting for other (operable) cases. In conclusion, our study demonstrates 1) the clinical efficacy of combination regimens with AGI and other anticancer agents in yielding clinically meaningful response rates and survival benefits, and 2) the utility of multi-analyte tumor profiling as a decision support system for clinicians to design personalized treatment strategies for patients with advanced or refractory cancers.

## Data Availability

The original contributions presented in the study are included in the article/Supplementary Material, further inquiries can be directed to the corresponding author.

## Glossary

AE: Adverse Event
AGI: Angiogenesis Inhibitor
ARC: advanced refractory cancers
BCR-ABL: fusion of ABL1 and BCR gene products (protein kinase)
c-KIT: Cellular Homolog of the Feline Sarcoma Viral Oncogene v-KIT
CNV: Copy Number Variations
CR: Complete Response
C-TACs: Circulating Tumor Associated Cells
CTCAE: Common Terminology Criteria for Adverse Events.
ctDNA: Circulating (cell-free) tumor DNA
DCR: Disease Control Rate
DGE: Differential Gene Expression
ETA: Encyclopedic Tumor Analysis
FGF: Fibroblast Growth Factor
FGFR: Fibroblast Growth Factor Receptor
IHC: Immunohistochemistry
ICC: Immunocytochemistry
mPFS: median Progression Free Survival
mOS: median Overall Survival
NGS: Next Generation Sequencing
ORR: Objective Response Rate
OS: Overall Survival
PDGF: Platelet Derived Growth Factor
PDGFR: Platelet Derived Growth Factor Receptor
PD: Progressive Disease (Disease Progression)
PFS: Progression Free Survival
PR: Partial Response
RAF: Rapidly Accelerated Fibrosarcoma (protein kinase)
RCT: Randomised Clinical Trials
RET: Rearranged during Transfection (protein kinase)
ReSP: Resistance and Sensitivity Profiling
RECIST: Response Evaluation Criteria in Solid Tumors
SD: Stable Disease
SNV: Single Nucleotide Variations
SoC: Standard of Care
TDCs: Tumor Tissue Derived Cells
USFDA: United States Food and Drug Administration
VEGF: Vascular Endothelial Growth Factor
VEGFR: Vascular Endothelial Growth Factor Receptor
