# Supplementary material for: Angiogenesis Inhibitors in Personalized Combination Regimens for the Treatment of Advanced Refractory Cancers
Source: Front Mol Med. 2021 Sep 20;1:749283. doi: 10.3389/fmmed.2021.749283 (PMC11285706; doi:10.3389/fmmed.2021.749283)
Supplement: Supplementary file 1 [file Table4.pdf]

Supplementary Table S04. Patient-wise and Overall Treatment Related Adverse Events

Patient ID	16740	23223	14173	21433	17463	18802	17782	15297	14522	25653	15188	21705	31918	20273	16425	18096	14851	15003	15648	27548	16387	17976	17425	32061	25760	31754	13304	12657	14402	14364	15852	15610	18102	14711	14264	17345	17984	16972	14252	14278	18093	14295	15730	15205	14461	18038	15777	18617	21833	12089	14552	14137	17488	14355	14656	16553	15902	28854	13299	12585						
Group	AGI_C	AGI_C	AGI_C	AGI_C	AGI_C	AGI_C	AGI_C	AGI_C	AGI_C	AGI_C	AGI_C	AGI_C	AGI_C	AGI_C	AGI_C	AGI_C	AGI_C	AGI_C	AGI_C	AGI_C	AGI_C	AGI_C	AGI_C	AGI_C	AGI_C	AGI_C	AGI_C	AGI_C	AGI_C	AGI_C	AGI_C	AGI_C	AGI_C	AGI_C	AGI_C	AGI_C	AGI_C	AGI_C	AGI_C	AGI_C	AGI_C	AGI_C	AGI_C	AGI_CT	AGI_CT	AGI_CT	AGI_CT	AGI_CT	AGI_CT	AGI_CT	AGI_CT	AGI_CT	AGI_CT	AGI_T	AGI_T	AGI_T	AGI_T	AGI_T	AGI_T	AGI_T	Overall		AGI_C		AGI_T±C	
**Any Grade**																																																													#	%	#	%	#	%
Anemia	-	-	-	-	-	Y	-	-	-	-	Y	-	-	-	-	-	-	-	Y	-	-	-	-	-	-	-	-	Y	-	-	-	-	-	-	-	Y	-	-	-	-	-	-	-	-	-	-	-	-	-	-	-	-	Y	-	Y	-	Y	-	-	Y	9	15.0%	5	11.6%	4	23.5%
Anorexia	Y	Y	Y	Y	Y	-	Y	-	Y	-	Y	Y	-	Y	Y	Y	-	Y	Y	Y	Y	-	-	-	Y	Y	Y	Y	-	Y	-	-	-	Y	Y	-	-	Y	Y	Y	-	Y	Y	Y	Y	Y	Y	Y	Y	-	Y	Y	Y	Y	Y	-	-	-	Y	Y	41	68.3%	28	65.1%	13	76.5%
Constipation	Y	-	Y	-	-	-	-	Y	-	-	-	-	-	Y	-	-	-	-	-	-	-	Y	-	-	-	-	Y	-	-	-	-	-	-	-	-	-	-	-	Y	-	-	-	-	-	-	-	-	-	-	-	-	-	-	Y	-	-	-	-	Y	-	9	15.0%	7	16.3%	2	11.8%
Diarrhoea	-	Y	Y	-	-	-	-	Y	-	-	-	-	-	-	-	-	-	-	-	-	-	-	-	-	Y	-	-	-	Y	Y	-	-	-	-	Y	-	-	-	-	-	-	-	-	-	Y	-	Y	-	Y	-	-	Y	-	-	Y	-	Y	-	-	-	13	21.7%	7	16.3%	6	35.3%
Edema	-	-	-	-	-	-	-	-	-	-	Y	-	Y	Y	Y	-	-	-	-	-	-	-	-	-	-	-	Y	-	-	-	Y	-	-	-	-	-	-	-	-	-	-	Y	-	-	-	-	-	Y	-	-	-	-	-	-	-	-	Y	-	Y	Y	11	18.3%	7	16.3%	4	23.5%
Fatigue	-	Y	Y	Y	-	Y	-	Y	Y	Y	Y	Y	Y	Y	Y	Y	Y	Y	Y	Y	Y	-	Y	Y	Y	Y	Y	-	Y	Y	Y	Y	Y	Y	Y	Y	Y	Y	Y	Y	Y	Y	Y	Y	Y	Y	Y	Y	Y	Y	Y	Y	Y	Y	Y	Y	-	Y	Y	Y	54	90.0%	38	88.4%	16	94.1%
Hyper-/Hypotension	-	-	-	-	-	-	-	-	-	-	-	-	-	Y	-	-	-	-	-	-	-	-	-	-	-	-	Y	-	-	Y	-	-	-	-	Y	-	-	-	-	-	-	-	-	-	-	-	-	-	-	-	-	Y	-	-	-	-	-	-	-	-	5	8.3%	4	9.3%	1	5.9%
Mucositis Oral	-	-	-	Y	-	Y	Y	-	-	-	-	-	-	Y	-	Y	-	Y	Y	-	-	Y	-	-	Y	-	-	-	Y	-	Y	-	Y	Y	-	Y	-	-	Y	Y	-	Y	Y	-	Y	-	-	-	-	Y	-	Y	Y	Y	Y	-	Y	-	Y	Y	27	45.0%	18	41.9%	9	52.9%
Myalgia	-	-	Y	-	Y	-	-	-	-	-	-	-	-	-	Y	-	-	-	-	-	-	-	-	-	-	-	Y	Y	-	-	-	-	-	-	-	-	-	-	-	Y	Y	-	Y	-	Y	-	-	-	Y	Y	-	-	-	Y	Y	-	-	-	-	Y	14	23.3%	8	18.6%	6	35.3%
Nausea	-	-	Y	-	-	-	Y	-	-	Y	-	Y	-	-	-	-	-	Y	-	Y	-	-	-	-	-	-	Y	Y	Y	-	-	-	-	-	-	-	-	-	-	Y	Y	Y	Y	Y	Y	-	Y	-	-	-	-	-	-	Y	-	Y	Y	-	Y	Y	21	35.0%	13	30.2%	8	47.1%
Neutropenia	-	Y	Y	Y	-	-	-	-	-	Y	-	Y	-	Y	-	-	-	-	-	-	-	-	-	-	-	-	-	Y	Y	-	-	Y	Y	-	Y	Y	-	-	Y	Y	-	-	-	-	-	-	-	-	-	Y	-	-	-	Y	-	-	-	-	Y	-	17	28.3%	14	32.6%	3	17.6%
Peripheral neuropathy	-	-	-	Y	-	-	-	-	-	-	-	-	-	-	-	-	-	-	-	Y	-	Y	-	-	-	Y	-	-	-	-	-	-	-	-	-	-	-	-	-	-	-	-	-	-	-	-	-	-	Y	-	-	-	-	-	-	-	-	-	-	Y	6	10.0%	4	9.3%	2	11.8%
Skin Disorders	-	-	Y	Y	-	-	-	-	-	-	-	-	-	-	-	-	-	-	-	-	-	-	-	-	-	-	-	-	-	-	-	-	-	-	Y	Y	-	-	Y	-	-	Y	-	-	-	-	-	-	-	-	-	Y	-	-	Y	Y	-	Y	-	-	10	16.7%	6	14.0%	4	23.5%
Pyrexia	-	-	Y	Y	-	-	-	Y	-	-	Y	Y	-	Y	-	Y	-	-	Y	-	-	Y	-	-	-	-	Y	Y	Y	-	-	-	-	-	-	-	-	-	-	-	-	-	-	-	-	-	-	-	-	-	-	Y	-	-	-	-	Y	-	Y	-	15	25.0%	12	27.9%	3	17.6%
Thrombocytopenia	-	-	Y	-	Y	-	-	-	-	-	Y	-	Y	-	-	Y	-	-	-	-	-	-	-	-	-	-	-	Y	-	-	-	Y	-	-	Y	-	-	-	-	-	-	-	-	-	-	-	-	-	Y	-	-	-	-	-	-	-	Y	-	Y	Y	12	20.0%	8	18.6%	4	23.5%
Vomiting	-	-	-	-	-	-	-	Y	-	Y	-	-	Y	-	-	-	-	-	-	-	-	-	-	-	-	-	Y	-	-	-	-	-	-	-	-	-	-	-	-	Y	-	Y	-	Y	-	-	-	-	-	-	Y	Y	-	Y	-	-	-	-	Y	-	11	18.3%	6	14.0%	5	29.4%
**Grade III**																																																													#	%	#	%	#	%
Anemia	-	-	-	-	-	Y	-	-	-	-	Y	-	-	-	-	-	-	-	-	-	-	-	-	-	-	-	-	-	-	-	-	-	-	-	-	-	-	-	-	-	-	-	-	-	-	-	-	-	-	-	-	-	Y	-	-	-	Y	-	-	-	4	6.7%	2	4.7%	2	11.8%
Anorexia	-	Y	-	-	-	-	-	-	-	-	-	-	-	Y	Y	-	-	-	-	-	-	-	-	-	-	-	-	-	-	-	-	-	-	-	-	-	-	-	-	Y	-	-	-	Y	-	-	-	-	Y	-	-	Y	-	Y	-	-	-	-	Y	-	9	15.0%	4	9.3%	5	29.4%
Constipation	-	-	-	-	-	-	-	-	-	-	-	-	-	-	-	-	-	-	-	-	-	-	-	-	-	-	Y	-	-	-	-	-	-	-	-	-	-	-	-	-	-	-	-	-	-	-	-	-	-	-	-	-	-	-	-	-	-	-	Y	-	2	3.3%	1	2.3%	1	5.9%
Diarrhoea	-	Y	-	-	-	-	-	-	-	-	-	-	-	-	-	-	-	-	-	-	-	-	-	-	-	-	-	-	-	-	-	-	-	-	-	-	-	-	-	-	-	-	-	-	-	-	Y	-	-	-	-	-	-	-	-	-	-	-	-	-	2	3.3%	1	2.3%	1	5.9%
Edema	-	-	-	-	-	-	-	-	-	-	-	-	-	Y	Y	-	-	-	-	-	-	-	-	-	-	-	-	-	-	-	-	-	-	-	-	-	-	-	-	-	-	-	-	-	-	-	-	-	-	-	-	-	-	-	-	-	Y	-	-	-	3	5.0%	2	4.7%	1	5.9%
Fatigue	-	Y	-	Y	-	Y	-	-	Y	Y	-	Y	-	Y	-	Y	-	Y	-	Y	-	-	Y	Y	Y	Y	-	-	-	-	-	-	Y	-	Y	Y	-	y	-	Y	Y	-	-	Y	Y	-	-	Y	Y	-	-	Y	-	Y	Y	-	-	Y	-	-	28	46.7%	20	46.5%	8	47.1%
Hyper-/Hypotension	-	-	-	-	-	-	-	-	-	-	-	-	-	-	-	-	-	-	-	-	-	-	-	-	-	-	-	-	-	Y	-	-	-	-	Y	-	-	-	-	-	-	-	-	-	-	-	-	-	-	-	-	Y	-	-	-	-	-	-	-	-	3	5.0%	2	4.7%	1	5.9%
Mucositis Oral	-	-	-	-	-	-	-	-	-	-	-	-	-	Y	-	-	-	Y	-	-	-	Y	-	-	-	-	-	-	-	-	-	-	-	-	-	-	-	-	Y	-	-	-	Y	-	-	-	-	-	-	-	-	Y	-	Y	-	-	-	-	Y	Y	9	15.0%	5	11.6%	4	23.5%
Myalgia	-	-	-	-	Y	-	-	-	-	-	-	-	-	-	-	-	-	-	-	-	-	-	-	-	-	-	Y	-	-	-	-	-	-	-	-	-	-	-	-	-	-	-	-	-	-	-	-	-	-	-	-	-	-	Y	-	-	-	-	-	Y	4	6.7%	2	4.7%	2	11.8%
Nausea	-	-	-	-	-	-	-	-	-	-	-	-	-	-	-	-	-	-	-	-	-	-	-	-	-	-	-	-	-	-	-	-	-	-	-	-	-	-	-	-	-	-	-	-	-	-	Y	-	-	-	-	-	-	Y	-	-	-	-	Y	Y	4	6.7%	0	0.0%	4	23.5%
Neutropenia	-	-	Y	-	-	-	-	-	-	-	-	-	-	-	-	-	-	-	-	-	-	-	-	-	-	-	-	-	Y	-	-	-	-	-	Y	-	-	-	-	-	-	-	-	-	-	-	-	-	-	-	-	-	-	Y	-	-	-	-	Y	-	5	8.3%	3	7.0%	2	11.8%
Peripheral neuropathy	-	-	-	Y	-	-	-	-	-	-	-	-	-	-	-	-	-	-	-	-	-	-	-	-	-	-	-	-	-	-	-	-	-	-	-	-	-	-	-	-	-	-	-	-	-	-	-	-	-	-	-	-	-	-	-	-	-	-	-	Y	2	3.3%	1	2.3%	1	5.9%
Skin Disorders	-	-	-	-	-	-	-	-	-	-	-	-	-	-	-	-	-	-	-	-	-	-	-	-	-	-	-	-	-	-	-	-	-	-	-	-	-	-	-	-	-	-	-	-	-	-	-	-	-	-	-	Y	-	-	-	-	-	Y	-	-	2	3.3%	0	0.0%	2	11.8%
Pyrexia	-	-	-	-	-	-	-	-	-	-	Y	-	-	-	-	-	-	-	-	-	-	-	-	-	-	-	-	-	Y	-	-	-	-	-	-	-	-	-	-	-	-	-	-	-	-	-	-	-	-	-	-	-	-	-	-	-	-	-	-	-	2	3.3%	2	4.7%	0	0.0%
Thrombocytopenia	-	-	Y	-	Y	-	-	-	-	-	-	-	-	-	-	Y	-	-	-	-	-	-	-	-	-	-	-	Y	-	-	-	Y	Y	-	Y	-	-	-	-	-	-	-	-	-	-	-	-	-	Y	-	-	-	-	-	-	-	Y	-	Y	-	10	16.7%	7	16.3%	3	17.6%
Vomiting	-	-	-	-	-	-	-	-	-	-	-	-	-	-	-	-	-	-	-	-	-	-	-	-	-	-	-	-	-	-	-	-	-	-	-	-	-	-	-	-	-	-	-	-	-	-	-	-	-	-	-	-	-	Y	-	-	-	-	-	-	1	1.7%	0	0.0%	1	5.9%
