# Supplementary material for: Angiogenesis Inhibitors in Personalized Combination Regimens for the Treatment of Advanced Refractory Cancers
Source: Front Mol Med. 2021 Sep 20;1:749283. doi: 10.3389/fmmed.2021.749283 (PMC11285706; doi:10.3389/fmmed.2021.749283)
Supplement: Supplementary file 2 [file Table1.pdf]

Supplementary Table S01. Demographics of Study Cohort

ID	Study	Age	Gender	Primary (Subtype)	Prior Systemic Lines	Prior Surgeries	Prior Radiation
12089	Non-Trial	53	M	Kidney (RCC)	3	Y	Y
12585	Non-Trial	65	F	Breast (IDC)	7	-	Y
12657	Non-Trial	22	F	Breast (IDC)	3	Y	Y
13299	Non-Trial	54	F	Ovary (AD)	2	Y	Y
13304	Non-Trial	51	F	Ovary (AD)	2	Y	Y
15188	Trial: LI	42	F	Sarcoma	3	Y	Y
16387	Trial: LI	68	F	Stomach (AD)	4	N	N
17425	Trial: LI	70	M	Pancreas (AD)	2	Y	N
17976	Trial: LI	50	M	Testes (GCT)	1	Y	N
18802	Trial: LI	43	M	Head and Neck (SCC)	2	Y	N
20273	Trial: LI	57	F	Breast (IDC)	3	Y	N
21433	Trial: LI	49	F	Occult (NET)	1	Y	N
21705	Trial: LI	38	F	Ovary (AD)	4	Y	N
21833	Trial: LI	55	F	Breast (IDC)	6	Y	Y
23223	Trial: LI	70	M	Liver (HCC)	2	N	N
25653	Trial: LI	48	M	Lung (AD)	3	N	N
25760	Trial: LI	31	F	Ovary (AD)	2	Y	N
27548	Trial: LI	40	F	Ovary (AD)	3	Y	N
28854	Trial: LI	39	M	Colorectum (AD)	3	N	Y
31754	Trial: LI	42	M	Esophagus (SCC)	2	N	Y
31918	Trial: LI	38	F	Ovary (AD)	2	Y	N
32061	Trial: LI	63	M	Colorectum (AD)	3	N	N
14137	Trial: RS	35	F	Cervix (NET)	3	Y	N
14173	Trial: RS	62	M	Lung (SCC)	2	N	N
14252	Trial: RS	51	M	Head and Neck (SCC)	4	Y	Y
14264	Trial: RS	59	F	Kidney (RCC)	1	Y	Y
14278	Trial: RS	52	M	Duodenum (NET)	2	Y	N
14295	Trial: RS	55	F	Head and Neck (SCC)	2	Y	Y
14355	Trial: RS	39	F	Breast (IDC)	4	Y	Y
14364	Trial: RS	52	M	Stomach (AD)	1	N	N
14402	Trial: RS	69	M	Stomach (AD)	2	Y	N
14461	Trial: RS	61	F	Ovary (AD)	7	Y	N
14522	Trial: RS	43	M	Colorectum (AD)	2	Y	Y
14552	Trial: RS	50	M	Head and Neck (SCC)	2	N	N
14656	Trial: RS	61	F	Breast (IDC)	5	Y	Y
14711	Trial: RS	46	F	Melanoma	4	Y	N
14851	Trial: RS	39	F	Colorectum (AD)	2	Y	N
15003	Trial: RS	69	F	Pancreas (AD)	1	Y	Y
15205	Trial: RS	46	F	Breast (IDC)	4	Y	Y
15297	Trial: RS	43	M	Kidney (RCC)	1	N	N
15610	Trial: RS	39	F	Breast (IDC)	7	Y	Y
15648	Trial: RS	51	M	Head and Neck (SCC)	2	Y	Y
15730	Trial: RS	66	F	Esophagus (SCC)	1	N	N
15777	Trial: RS	59	M	Pancreas (AD)	3	N	Y
15852	Trial: RS	46	M	Head and Neck (SCC)	2	Y	Y
15902	Trial: RS	47	F	Breast (IDC)	4	Y	Y
16425	Trial: RS	43	F	Pilomatrixoma	3	Y	Y
16553	Trial: RS	36	M	Head and Neck (SCC)	2	N	Y
16740	Trial: RS	62	M	Occult (NET)	1	N	N
16972	Trial: RS	50	M	Head and Neck (SCC)	1	Y	Y
17345	Trial: RS	43	M	Head and Neck (SCC)	3	Y	Y
17463	Trial: RS	45	F	Breast (IDC)	2	N	N
17488	Trial: RS	35	M	Head and Neck (SCC)	2	Y	Y
17782	Trial: RS	33	M	Colorectum (AD)	1	Y	N
17984	Trial: RS	47	M	Head and Neck (SCC)	1	N	N
18038	Trial: RS	28	F	Breast (IDC)	3	N	Y
18093	Trial: RS	45	M	Head and Neck (SCC)	3	N	Y
18096	Trial: RS	55	M	Stomach (AD)	3	Y	Y
18102	Trial: RS	46	M	Head and Neck (SCC)	2	Y	Y
18617	Trial: RS	71	F	Liver (HCC)	1	N	N

Breast (IDC)	11
Cervix (NET)	1
Colorectum (AD)	5
Duodenum (NET)	1
Esophagus (SCC)	2
Head and Neck (SCC)	13
Kidney (RCC)	3
Liver (HCC)	2
Lung (AD)	1
Lung (SCC)	1
Melanoma	1
Occult (NET)	2
Ovary (AD)	7
Pancreas (AD)	3
Pilomatrixoma	1
Sarcoma	1
Stomach (AD)	4
Testes (NSCGT)	1
Total	60

Male	30
Female	30

Median Age	49
Age Range	22 - 71

IDC: Invasive Ductal Carcinoma
NET: Neuroendocrine Tumor
AD: Adenocarcinoma
SCC: Squamous Cell Carcinoma
RCC: Renal Cell Carcinoma
HCC: Hepatocellular Carcinoma
GCT: Germ Cell Tumor
