# Supplementary material for: Angiogenesis Inhibitors in Personalized Combination Regimens for the Treatment of Advanced Refractory Cancers
Source: Front Mol Med. 2021 Sep 20;1:749283. doi: 10.3389/fmmed.2021.749283 (PMC11285706; doi:10.3389/fmmed.2021.749283)
Supplement: Supplementary file 3 [file Table2.pdf]

Supplementary Table 02. Patient-wise targetable indications and treatment regimen.

ID	SNV	CNV	GOE	ICC	IHC	Anti-Angiogenesis Targeted Agent	Other Targeted Agent	Endocrine Antaignist	Cytotoxic Agent	Regimen Details	Therapy Category
12089	FGFR, c-KIT	-	-	-	AR	Axitinib	-	Bicalutamide	5-fluorouracil, Methotrexate	Tab Axitnib, 5 mg, PO, 1-0-0<br>Tab Bicalutamide, 50 mg, PO, 0-1-0<br>IV 5-fluorouracil, 500 mg, D1 of 7D<br>IV Methotrexate, 25 mg, D1 of 7D	AGI_CT
12585	c-KIT	PTEN (-)	-	-	-	Axitinib	Temsirolimus	-	-	Tab Axitnib, 5 mg, PO, 1-0-0<br>IV Temsirolimus, 25 mg, D1 of 21D	AGI_T
12657	-	-	VEGFR, PDGFR, c-KIT	-	-	Axitinib	-	-	Carboplatin, Gemcitabine	Tab Axitnib, 5 mg, PO, 1-0-0<br>IV Carboplatin, 300 mg, D1 of 21D<br>IV Gemcitabine, 1000 mg, D1 of 21D,	AGI_C
13299	VEGFR	-	-	-	-	Axitinib	Temsirolimus	-	-	Tab Axitnib, 5 mg, PO, 1-0-0<br>IV Temsirolimus, 25 mg, D1 of 21D	AGI_T
13304	c-KIT	-	-	-	-	Axitinib	-	-	Docetaxel, Topotecan	Tab Axitnib, 5 mg, PO, 1-0-0<br>IV Docetaxel, 60 mg, D1 of 21D<br>IV Topotecan, 1mg, D1-D5 of 21D	AGI_C
14137	-	-	c-KIT, RET, EGFR, ERBB2	-	-	Axitinib	Erlotinib	-	Oxaliplatin	Tab Axitnib, 5 mg, PO, 1-0-0<br>Tab Erlotinib, 100 mg, PO, 0-1-0<br>IV Oxaliplatin, _200 mg, D1 of 21D	AGI_CT
14173	-	-	FGFR	-	-	Pazopanib	-	-	Vinblastine	Tab Pazopanib, 400 mg, PO, 0-1-0<br>IV Vinblastine, 9 mg, D1 of 7D	AGI_C
14252	-	c-KIT (+)	VEGFR, PDGFR, c-KIT	-	-	Axitinib	-	-	Cyclophosphamide, Methotrexate	Tab Axitnib, 5 mg, PO, 1-0-0<br>IV Cyclophosphamide 900 mg, D1 of 21D<br>IV Methotrexate, 25 mg, D1 of 21D	AGI_C
14264	-	-	c-KIT	-	-	Axitinib	-	-	Capecitabine	Tab Axitnib, 5 mg, PO, 1-0-0<br>Tab Capecitabine, 500 mg, PO, 0-1-0, 14D on/off	AGI_C
14278	-	-	VEGF	-	-	Axitinib	-	-	Epirubicin	Tab Axitnib, 5 mg, PO, 1-0-0<br>IV Epirubicin, 80 mg, D1 of 21D	AGI_C
14295	-	-	VEGFR, PDGFR	-	-	Axitinib	-	-	Cyclophosphamide, Methotrexate	Tab Axitnib, 5 mg, PO, 1-0-0<br>IV Cyclophosphamide 350 mg, D1 of 7D<br>IV Methotrexate, 30 mg, D1 of 7D	AGI_C
14355	PIK3CA	-	VEGF	-	-	Bevacizumab	Temsirolimus	-	-	IV Bevacizumab, 480 mg, D1 of 21D<br>IV Temsirolimus, 25 mg, D1 of 21D	AGI_T
14364	-	-	PDGFR	-	-	Axitinib	-	-	Docetaxel, Dacarbazine	Tab Axitnib, 5 mg, PO, 1-0-0<br>IV Docetaxel, 60 mg, D1 of 21D<br>IV Dacarbazine, 1000 mg, D1 of 21D	AGI_C
14402	-	-	PDGFR	-	-	Axitinib	-	-	Paclitaxel, Methotrexate	Tab Axitnib, 5 mg, PO, 1-0-0<br>IV Paclitaxel, 110 mg, D1 of 7D<br>IV Methotrexate, 25 mg, D1 of 7D	AGI_C
14461	-	-	VEGF	-	AR, ER	Bevacizumab	-	Tamoxifen, Bicalutamide	Mitoxantrone	IV Bevacizumab, 720 mg, D1 of 21D<br>Tab Tamoxifen, 20 mg, PO, 1-0-0<br>Tab Bicalutamide, 50 mg, PO, 0-1-0<br>IV Mitoxantrone, 15 mg, D1 of 21D	AGI_CT
14522	-	-	VEGFR	-	-	Regorafenib	-	-	Paclitaxel, Methotrexate	Tab Regorafenib, 40 mg, PO, 1-1-0<br>IV Paclitaxel, 110 mg, D1 of 21D<br>Tab Methotrexate, 2.5 mg, PO, 6-0-0, D1 of 7D	AGI_C

ID	SNV	CNV	GOE	ICC	IHC	Anti-Angiogenesis Targeted Agent	Other Targeted Agent	Endocrine Antaignist	Cytotoxic Agent	Regimen Details	Therapy Category
14552	-	-	PDGFR, EGFR	-	-	Axitinib	Cetuximab	-	Cisplatin	Tab Axitnib, 5 mg, PO, 1-0-0<br>IV Cetuximab, 100 mg, D1 of 7D<br>IV Cisplatin, 40 mg, D1 of 7D	AGI_CT
14656	PIK3CA	-	VEGF	-	HER2	Bevacizumab	Everolimus, Lapatinib	-	-	IV Bevacizumab, 400 mg, D1 of 21D<br>Tab Everolimus, 5 mg, PO, 0-1-0 (alternate days)<br>Tab Lapatinib, 250 mg, PO, 0-0-1 (alternate days)	AGI_T
14711	-	-	PDGFR	-	-	Axitinib	-	-	Vincristine, Etoposide	Tab Axitnib, 5 mg, PO, 1-0-0<br>IV Vincristine,1 mg, D1 of 7D<br>Tab Etoposide, 50 mg, PO, 0-1-0, 7D on/off	AGI_C
14851	KRAS	-	-	-	-	Regorafenib	-	-	Etoposide, Methotrexate	Tab Regorafenib, 40 mg, PO, 0-1-0<br>Tab Etoposide, 50 mg, PO, 0-1-0, 7D on/off<br>Tab Methotrexate, 2.5 mg, PO, 6-0-0, D1 of 7D	AGI_C
15003	-	-	FGFR	-	-	Regorafenib	-	-	Docetaxel, Cyclophosphamide	Tab Regorafenib, 40 mg, PO, 0-1-0<br>IV Docetaxel, 100 mg, D1 of 21D<br>IV Cyclophosphamide 800 mg, D1 of 21D	AGI_C
15188	-	-	PDGFR	-	-	Axitinib	-	-	Vinorelbine, Methotrexate	Tab Axitnib, 5 mg, PO, 1-0-0<br>IV Vinorelbine, 15 mg, D1, D8 of 21D<br>IV Methotrexate, 30 mg, D1, D8 of 21D	AGI_C
15205	-	GPR124 (+)	-	-	ER	Bevacizumab	-	Fulvestrant	Eribulin	IV Bevacizumab, 500 mg, D1, D8 of 21D<br>Inj Fulvestrant, 500 mg, IM, D1 of 21D<br>IV Eribulin, 1 mg, D1 of 21D	AGI_CT
15297	-	-	VEGF	-	-	Bevacizumab	-	-	5-fluorouracil, Irinotecan	IV Bevacizumab, 600 mg, D1 of 21D<br>IV 5-fluorouracil, 1000 mg, D1 of 21D<br>IV Irinotecan, 240 mg, D1 of 21D	AGI_C
15610	PDGFR	-	-	-	-	Axitinib	-	-	Gemcitabine, Dacarbazine	Tab Axitnib, 5 mg, PO, 1-0-0<br>IV Gemcitabine, 800 mg, D1 of 21D,<br>IV Dacarbazine, 800 mg, D1 of 21D	AGI_C
15648	-	-	PDGFR	-	-	Imatinib	-	-	Docetaxel, Gemcitabine	Tab Imatinib, 400 mg, PO, 0-1-0<br>IV Docetaxel, 60 mg, D1 of 21D<br>IV Gemcitabine, 1000 mg, D1 of 21D,	AGI_C
15730	-	-	VEGFR, VEGF	-	-	Axitinib	-	-	Etoposide	Tab Axitnib, 5 mg, PO, 1-0-0<br>Tab Etoposide, 50 mg, PO, 0-1-0, 7D on/off	AGI_C
15777	EGFR	-	VEGF	-	-	Bevacizumab	Afatinib	-	Etoposide	IV Bevacizumab, 400 mg, D1 of 14D<br>Tab Afatinib, 40 mg, PO, 1-0-0<br>Tab Etoposide, 50 mg, PO, 0-1-0, 7D on/off	AGI_CT
15852	-	-	VEGFR	-	-	Axitinib	-	-	Cyclophosphamide, Eribulin	Tab Axitnib, 5 mg, PO, 1-0-0<br>IV Cyclophosphamide 900 mg, D1 of 21D<br>IV Eribulin, 1 mg,	AGI_C
15902	PDGFR, PIK3CA	-	-	-	-	Pazopanib	Everolimus	-	-	Tab Pazopanib, 400 mg, PO, 0-1-0<br>Tab Everolimus, 5 mg, PO, 0-1-0	AGI_T
16387	-	-	-	VEGF	-	Bevacizumab	-	-	Carboplatin, Doxorubicin	IV Bevacizumab, 200 mg, D1 of 21D<br>IV Carboplatin, 270 mg, D1 of 21D<br>IV Doxorubicin, 30 mg, D1 of 21D	AGI_C
16425	-	-	VEGF	-	-	Bevacizumab	-	-	5-fluorouracil, Etoposide	IV Bevacizumab, 480 mg, D1 of 21D<br>IV 5-fluorouracil, 800 mg, D1 of 21D<br>Tab Etoposide, 50 mg, PO, 1-0-0 7D on/off	AGI_C

ID	SNV	CNV	GOE	ICC	IHC	Anti-Angiogenesis Targeted Agent	Other Targeted Agent	Endocrine Antaignist	Cytotoxic Agent	Regimen Details	Therapy Category
16553	-	EGFR (+)	-	-	-	Bevacizumab	Erlotinib	-	-	IV Bevacizumab, 500 mg, D1 of 21D<br>Tab Erlotinib, 100 mg, PO, 0-1-0	AGI_T
16740	-	-	VEGFR, PDGFR	-	-	Axitinib	-	-	Etoposide, Cabazitaxel	Tab Axitnib, 5 mg, PO, 1-0-0<br>IV Etoposide, 120 mg, D1-D3 of 21D<br>IV Cabazitaxel, 16 mg, D1-D3 of 21D	AGI_C
16972	-	-	PDGFR	-	-	Axitinib	-	-	Methotrexate, Vinblastine	Tab Axitnib, 5 mg, PO, 1-0-0<br>IV Methotrexate, 25 mg, D1 of 7D<br>IV Vinblastine, 9 mg, D1 of 7D	AGI_C
17345	-	-	VEGFR, PDGFR	-	-	Axitinib	-	-	Topotecan, Doxorubicin	Tab Axitnib, 5 mg, PO, 1-0-0<br>IV Topotecan, 1mg, D1-D3 of 28D<br>IV Doxorubicin, 25 mg, D1-D3 of 28D	AGI_C
17425	-	-	-	VEGF	-	Bevacizumab	-	-	Cabazitaxel	IV Bevacizumab, 3-- mg, D1 of 21D<br>IV Cabazitaxel, 20 mg, D1 of 21D	AGI_C
17463	-	-	VEGF	-	-	Bevacizumab	-	-	Etoposide, Eribulin	IV Bevacizumab, 450 mg, D1,D8 of 21D<br>Tab Etoposide, 50 mg, PO, 0-1-0, 7D on/off<br>IV Eribulin, 1 mg, D1,D8 of 21D	AGI_C
17488	-	-	PDGFR	-	-	Axitinib	Everolimus	-	Methotrexate	Tab Axitnib, 5 mg, PO, 1-0-0<br>Tab Everolimus, 5 mg, PO, 0-1-0<br>IV Methotrexate, 25 mg, D1 of 7D	AGI_CT
17782	-	-	PDGFR	-	-	Sunitinib	-	-	Gemcitabine, Irinotecan	Cap Sunitinib, 12.5 mg, PO, 0-1-0<br>IV Gemcitabine, 800 mg, D1, D8 of 21D,<br>IV Irinotecan, 170 mg, D1 of 21D	AGI_C
17976	-	-	-	VEGF	-	Bevacizumab	-	-	Paclitaxel, Epirubicin	IV Bevacizumab, 580 mg, D1 of 21D<br>IV Paclitaxel, 280 mg, D1 of 21D<br>IV Epirubicin, 85 mg, D1 of 21D	AGI_C
17984	-	-	PDGFR	-	-	Axitinib	-	-	Etoposide, Methotrexate	Tab Axitnib, 5 mg, PO, 1-0-0<br>IV Etoposide 150 mg, D1-D3 of 21D<br>IV Methotrexate, 100 mg, D1 of 21D	AGI_C
18038	-	-	VEGF	-	ER, AR	Bevacizumab	-	Tamoxifen, Bicalutamide	Eribulin	IV Bevacizumab, 500 mg, D1 of 21D<br>Tab Tamoxifen, 20 mg, PO, 1-0-0<br>Tab Bicalutamide, 50 mg, PO, 0-1-0<br>IV Eribulin, 1 mg, D1,D8 of 21D	AGI_CT
18093	-	-	PGDFR	-	-	Axitinib	-	-	5-fluoroauracil, Eribulin	Tab Axitnib, 5 mg, PO, 1-0-0<br>IV 5-fluorouracil, 250 mg, D1,D8 of 21D<br>IV Eribulin, 1 mg, D1,D8 of 21D	AGI_C
18096	-	FGFR (+)	-	-	-	Pazopanib	-	-	Gemcitabine, Pemetrexed	Tab Pazopanib, 400 mg, PO, 0-1-0<br>IV Gemcitabine, 800 mg, D1,D8 of 21D,<br>IV Pemetrexed, 400 mg, D1 of 21D	AGI_C
18102	-	-	PDGFR	-	-	Axitinib	-	-	Irinotecan, Vinorelbine	Tab Axitnib, 5 mg, PO, 1-0-0<br>IV Irinotecan, 100 mg, D1,D8 of 21D,<br>IV Vinorelbine, 20 mg, D1,D8 of 21D	AGI_C
18617	-	-	VEGF	-	AR	Bevacizumab	-	Bicalutamide	Gemcitabine, Vinorelbine	IV Bevacizumab, 360 mg, D1 of 14D<br>Tab Bicalutamide, 50 mg, PO, 0-1-0<br>IV Gemcitabine, 1000 mg, D1, D8 of 21D,<br>IV Vinorelbine, 20 mg, D1, D8 of 21D,	AGI_CT

ID	SNV	CNV	GOE	ICC	IHC	Anti-Angiogenesis Targeted Agent	Other Targeted Agent	Endocrine Antaignist	Cytotoxic Agent	Regimen Details	Therapy Category
18802	-	-	-	VEGFR	-	Axitinib	-	-	Paclitaxel, Temozolomide	Tab Axitnib, 5 mg, PO, 1-0-0<br>IV Paclitaxel, 130 mg, D1, D15 of 21D<br>IV Temozolomide, 100 mg, D1 of 21D	AGI_C
20273	-	-	-	VEGFR, EGFR	-	Axitinib, Bevacizumab	-	-	Epirubicin	Tab Axitnib, 5 mg, PO, 1-0-0<br>IV Bevacizumab, 500 mg, D1 of 21D<br>IV Epirubicin, 70 mg, D1 of 21D	AGI_C
21433	-	-	-	VEGFR	-	Axitinib	-	-	Gemcitabine, Cisplatin	Tab Axitnib, 5 mg, PO, 1-0-0<br>IV Gemcitabine, 600 mg, D1,D8 of 21D,<br>IV Cisplatin, 80 mg, D1,D8 of 21D,	AGI_C
21705	-	-	PDGFR	-	-	Axitinib	-	-	Vinorelbine	Tab Axitnib, 5 mg, PO, 1-0-0<br>IV Vinorelbine, 25 mg, D1,D8 of 21D	AGI_C
21833	-	FGFR (+)	-	ER	-	Pazopanib	-	Fulvestrant	Doxorubicin	Tab Pazopanib, 400 mg, PO, 0-1-0<br>Inj Fulvestrant, 500 mg, IM, D1 of 21D<br>IV Doxorubicin, 50 mg, D1 of 21D	AGI_CT
23223	-	-	-	VEGFR	-	Axitinib	-	-	Epirubicin, Paclitaxel	Tab Axitnib, 5 mg, PO, 1-0-0<br>IV Epirubicin, 50 mg, D1 of 21D,<br>IV Paclitaxel, 130 mg, D1 of 21D	AGI_C
25653	-	-	-	VEGFR	-	Axitinib	-	-	5-fluorouracil, Irinotecan	Tab Axitnib, 5 mg, PO, 1-0-0<br>IV 5-fluorouracil, 540 mg, D1 of 21D<br>IV Irinotecan, 160 mg, D1 of 21D	AGI_C
25760	-	-	-	VEGF	-	Bevacizumab	-	-	Docetaxel, Oxaliplatin	IV Bevacizumab, 500 mg, D1 of 21D<br>IV Docetaxel, 55 mg, D1 of 21D<br>IV Oxaliplatin, 65 mg, D1 of 21D	AGI_C
27548	-	-	-	VEGF	-	Bevacizumab	-	-	Paclitaxel	IV Bevacizumab, 500 mg, D1 of 21D<br>IV NAB-Paclitaxel, 105 mg, D1 of 21D	AGI_C
28854	-	-	-	VEGFR, EGFR	-	Regorafenib	Cetuximab	-	-	Tab Regorafenib, 400 mg, PO, 0-1-0<br>IV Cetuximab, 350 mg, D1 of 7D	AGI_T
31754	-	-	-	VEGF	-	Bevacizumab	-	-	Methotrexate, Vinblastine	IV Bevacizumab, 300 mg, D1 of 21D<br>IV Methotrexate, 35 mg, D1,D8 of 21D<br>IV Vinblastine, 8 mg, D1 of 7D	AGI_C
31918	-	-	-	VEGFR	-	Axitinib	-	-	5-fluorouracil, Mitomycin	Tab Axitnib, 5 mg, PO, 1-0-0<br>IV 5-fluorouracil, 1600 mg, D1 of 28D<br>IV Mitomycin, 8 mg, D1-D4 of 28D	AGI_C
32061	-	-	-	VEGFR	-	Regorafenib	-	-	Gemcitabine, Oxaliplatin	Tab Regorafenib, 40 mg, PO, 0-1-0<br>IV Gemcitabine, 1000 mg, D1,D15 of 21D,<br>IV Oxaliplatin, 130 mg, D1,D15 of 21D	AGI_C

SNV	Single Nucleotide Variation
CNV	Copy Number Variation (+) = gain, (-) = loss
GOE	Gene OverExpression (RNA)
ICC	Immunocytochemistry
IHC	Immunohistochemistry

Blue text indicates non-Angiogenesis related indications
