# Supplementary material for: Angiogenesis Inhibitors in Personalized Combination Regimens for the Treatment of Advanced Refractory Cancers
Source: Front Mol Med. 2021 Sep 20;1:749283. doi: 10.3389/fmmed.2021.749283 (PMC11285706; doi:10.3389/fmmed.2021.749283)
Supplement: Supplementary file 4 [file Table3.pdf]

Supplementary Table S03. Patient-wise Treatment Outcomes

ID	Category	Response	Prior PFS (PFS1) (months)	PFS in Study (PFS2) (months)	OS (months)	PFS2:PFS1
12657	AGI_C	PR	1.0	4.0	5.0	4.0
13304	AGI_C	PR	4.0	2.0	2.0	0.5
14173	AGI_C	SD	4.7	9.5	13.7	2.0
14252	AGI_C	SD	3.0	12.1	19.9	4.0
14264	AGI_C	SD	1.2	11.0	19.8	9.2
14278	AGI_C	SD	4.2	11.5	13.8	2.7
14295	AGI_C	SD	2.3	11.9	16.1	5.2
14364	AGI_C	SD	5.8	5.5	5.5	0.9
14402	AGI_C	SD	1.8	8.6	8.6	4.8
14522	AGI_C	PR	7.5	10.5	14.6	1.4
14711	AGI_C	PR	49.1	10.5	21.7	0.2
14851	AGI_C	SD	5.5	2.6	2.6	0.5
15003	AGI_C	PR	15.5	3.5	16.2	0.2
15188	AGI_C	SD	3.0	3.6	3.6	1.2
15297	AGI_C	SD	2.4	3.4	8.0	1.4
15610	AGI_C	PR	2.5	3.2	3.2	1.3
15648	AGI_C	SD	36.0	4.5	4.5	0.1
15730	AGI_C	SD	3.2	6.9	12.1	2.2
15852	AGI_C	SD	2.7	6.0	6.0	2.2
16387	AGI_C	PD	2.0	1.8	5.1	0.9
16425	AGI_C	PR	8.3	5.9	29.4	0.7
16740	AGI_C	PR	1.0	2.1	2.1	2.1
16972	AGI_C	SD	1.9	3.2	9.2	1.7
17345	AGI_C	SD	2.8	4.9	7.1	1.8
17425	AGI_C	SD	3.0	4.4	8.8	1.5
17463	AGI_C	SD	1.5	2.6	11.5	1.7
17782	AGI_C	SD	3.0	4.3	19.1	1.4
17976	AGI_C	PR	6.0	12.9	14.9	2.2
17984	AGI_C	SD	1.7	4.2	27.8	2.5
18093	AGI_C	SD	14.7	4.3	4.3	0.3
18096	AGI_C	PR	7.3	3.9	19.0	0.5
18102	AGI_C	PR	4.0	3.8	6.6	1.0
18802	AGI_C	PR	4.0	6.2	6.2	1.6
20273	AGI_C	PR	5.0	4.2	4.5	0.8
21433	AGI_C	PR	2.0	6.1	11.5	3.1
21705	AGI_C	SD	5.0	5.3	12.3	1.1
23223	AGI_C	SD	1.0	3.6	7.7	3.6
25653	AGI_C	SD	3.0	3.7	19.1	1.2
25760	AGI_C	PD	3.0	2.8	2.8	0.9
27548	AGI_C	PR	3.0	9.7	17.9	3.2
31754	AGI_C	PD	3.0	2.3	4.7	0.8
31918	AGI_C	PR	3.0	6.2	8.9	2.1
32061	AGI_C	SD	4.0	5.2	8.0	1.3
12089	AGI_T±C	PR	1.0	4.0	4.0	4.0
14137	AGI_T±C	PR	6.0	4.5	4.5	0.8
14461	AGI_T±C	PR	5.4	12.5	12.5	2.3
14552	AGI_T±C	PR	5.1	6.7	6.7	1.3
15205	AGI_T±C	PR	2.7	9.0	19.0	3.3
15777	AGI_T±C	SD	10.8	7.5	9.0	0.7
17488	AGI_T±C	SD	2.9	4.9	7.8	1.7
18038	AGI_T±C	SD	4.0	3.9	10.0	1.0
18617	AGI_T±C	SD	14.3	4.1	4.1	0.3
21833	AGI_T±C	PR	4.0	12.9	14.2	3.2
12585	AGI_T±C	PR	4.6	5.0	16.2	1.1
13299	AGI_T±C	PR	3.0	2.0	2.0	0.7
14355	AGI_T±C	PR	1.7	6.7	14.6	3.9
14656	AGI_T±C	PR	12.1	10.6	22.9	0.9
15902	AGI_T±C	SD	5.7	15.2	15.2	2.7
16553	AGI_T±C	PR	8.9	5.7	8.3	0.6
28854	AGI_T±C	PR	3.0	11.7	17.0	3.9
