# Supplementary material for: Angiogenesis Inhibitors in Personalized Combination Regimens for the Treatment of Advanced Refractory Cancers
Source: Front Mol Med. 2021 Sep 20;1:749283. doi: 10.3389/fmmed.2021.749283 (PMC11285706; doi:10.3389/fmmed.2021.749283)
Supplement: Supplementary file 5 [file DataSheet1.docx]

**Tissue Collection**

Approximately 5 × 5 × 5 mm freshly biopsied tumor tissue was transferred into 5 mL transport medium (that preserved the viability of tumor cells) and stored at 4 °C during transit. Fresh tissue was processed within 12 hours of collection.

**Histopathology and Immunohistochemistry**

Formalin-Fixed Paraffin-Embedded (FFPE) blocks were prepared from freshly biopsied tumor tissue and evaluated by standard Histopathological Examination (HPE) to determine tumor content. Tissue samples with ≥80% tumor content were acceptable for molecular evaluations. Standard Immunohistochemistry (IHC) was performed on slides prepared from FFPE blocks to determine status of AR, ER, HER2, TOPO and TUBB.

**Harvesting of Viable Tumor Tissue Derived Cells (TDCs)**

Freshly biopsied tumor tissue with >70% tumor content were dissociated into single-cell suspensions by a combination of mechanical dissociation and enzymatic degradation of the extracellular matrix using the Tumor Cell Isolation Kit, human kit components and the gentleMACS™ Dissociator (Miltenyi Biotech, Germany). The single cell suspension of Tumor Derived Cells (TDCs) obtained by this method was cultured at 37^°^C under 5% CO_2_ and 4% O_2_ for 24 hours and viable TDCs were then harvested for further applications.

**Tumor DNA Isolation**

Genomic DNA was isolated from the FFPE tumor blocks using a GeneRead DNA FFPE kit (Qiagen, Germantown, USA) as per the manufacturer’s instructions. DNA was quantified at 260 nm and quality was determined by measuring the ratio of absorbance at 260/280 nm using a NanoDrop 2000 (Thermo Fisher Scientific, Waltham, USA).

**Tumor RNA Isolation**

Total tumor RNA was isolated using an mirVana miRNA isolation kit (Ambion, Austin, USA) as per the manufacturer’s instruction. Total RNA was quantified using a Qubit 2 Fluorometer (Thermo Fisher Scientific, Waltham, USA) with the manufacturer’s RNA assay kit.

**Blood Collection and Processing**

Approximately 10 mL peripheral blood was collected by venous puncture in each of the Cell-Free DNA BCT® and EDTA vacutainer tubes. Blood was stored and transported at 2-8°C. Plasma was separated by centrifugation at 3000× g for 20 min at 2-8°C, followed by 16000× g for 10 min at 20–25°C. Plasma from Cell-Free DNA BCT® tubes without hemolysis were processed for cell-free nucleic acid isolation.

**Cell-Free DNA (ctDNA) Isolation**

Total ctDNA was purified from 2 mL plasma using a Circulating Nucleic Acid kit (QIAGEN, Germantown, USA) as per the manufacturer’s protocol. ctDNA was quantified using an HS DNA Qubit assay (Life Technologies, Carlsad, USA).

**Exosomal RNA Isolation**

Plasma samples (2 ml) from EDTA tubes were centrifuged at 16000× g for 10 min at 4 °C and filtered via a 0.45 µm membrane to remove larger vesicles. The filtrate was used for the extraction of total exosomal RNA using an ExoRNeasy serum/plasma kit (QIAGEN, Germantown, USA) according to the manufacturer’s protocol [17]. Purified exosomal RNA was quantified using an miRNA Qubit assay (Life Technologies, Carlsad, USA).

**Harvest of Viable Circulating Tumor Associated Cells (C-TACs)**

C-TACs were enriched and harvested from Peripheral Blood Mononuclear Cells (PBMCs) using an epigenetically activating medium as described prviously^1^. PBMCs were treated with epigenetically activating media for up to 100 hours at 37°C under 5% CO_2_, 4% O_2_. The medium induces cell death in normal (non-malignant) cells with functional apoptotic machinery while simultaneously conferring survival privilege on apoptosis-resistant cells of tumorigenic origin, i.e. Circulating Tumor Associated Cells (C-TACs; EpCAM+, PanCK+, CD45±) and their clusters. C-TACs include CTCs (EpCAM+, PanCK+, CD45-) as well as other cell types such as Tumor Associated Macrophages (TAMs) and Tumor Associated Fibroblasts (TAFs).

**DNA Mutation Profiling**

Tumor DNA (20 ng) was used for NGS library preparation via PCR-based Ampliseq target enrichment protocol. Libraries of 100 pmol were sequenced using Ion Proton (Thermo Fisher Scientific, Waltham, USA). Torrent Suite™ v5.2 (Thermo Fisher Scientific, Waltham, USA) software was used to perform primary analysis, including signal processing and base calling. Primary QC parameters were: minimum read length of 25 bases, read quality trimming of 17 QV, window size for quality trimming 30 bp. The processed sequenced data were aligned to the reference genome GRCh37/hg19 to generate Binary Alignment/Map (BAM) files. Sequencing data were considered for downstream analysis with coverage at ≥10,000× depth and >80% amplicons with at least 600 reads. The aligned data were analyzed using Torrent Variant Caller software with optimized parameters such as minimum allele frequency (0.003), minimum mapping quality (4), minimum coverage (600), down sample to coverage (10,000) and position bias (1). Reported somatic variants of >0.5% allele frequency (AF) were compared to the reference genome hg19. The Integrative Genomics Viewer (IGV) was used to visualize the read alignment and the presence of variants against the reference genome and to confirm the veracity of the variant calls by checking for possible strand biases and sequencing errors. All the germline variants found in the 1000 Genomes Project or The Exome Aggregation Consortium (ExAC) with a frequency of >0.1% were excluded. All somatic mutations were annotated, sorted and interpreted using COSMIC and/or TCGA data. Variants with <0.5% AF were confirmed orthogonally with digital droplet polymerase chain reaction (ddPCR, BioRad) using the rare mutation assay as per the manufacturer’s protocol.

A 50-gene NGS panel () was used for ctDNA profiling and a 453-gene NGS panel () was used for tumor DNA profiling to detect somatic hotspot mutations reported at high frequency in multiple cancer types as identified from TCGA, COSMIC, ICGC, MD Anderson Cancer Center and My Cancer Genome databases.

**mRNA Profiling (Transcriptome Analysis)**

The Ion AmpliSeq™ Transcriptome Human Gene Expression Research Panel was used to determine the expression of 20,802 genes including 18,574 coding genes and 2228 non-coding genes based on University of California Santa Cruz (UCSC) hg19 annotation. Exosomal RNA from asymptomatic individuals (male) was used as a control for cancer exosomal mRNA analysis. RNA prepared from normal tissue was used as a control for tumor mRNA analysis. A barcoded cDNA library was generated with a SuperScript® VILO™ cDNA Synthesis kit from 20 ng of exosomal RNA. The cDNA was amplified using Ion AmpliSeq™ technology as per the manufacturer’s instructions (Thermo Fisher Scientific, Waltham, USA). Amplified cDNA libraries were evaluated for quality on a Bioanalyzer 2100E using a high sensitivity DNA 1000 chip (Agilent Technologies, Santa Clara, USA) and quantified using an Ion Library TaqMan™ Quantitation Kit (Thermo Fischer Scientific, Waltham, USA)/KAPA Library Quantification Kits (KAPA Biosystems / Roche, Basel, Switzerland). Pooled libraries of 100 pM were amplified using emulsion PCR on an Ion Torrent OneTouch2 and enriched as per the manufacturer’s instructions. Templated libraries were sequenced on an Ion Torrent Proton™ sequencing system, using an Ion PI sequencing kit and an Ion PI chip (Thermo Fisher Scientific, Waltham, USA). Analysis of AmpliSeq RNA sequencing data was performed using the AmpliSeq-RNA plugin available for Ion Torrent sequencing platforms. This plugin uses the Torrent Mapping Alignment Program (TMAP—https://github.com/iontorrent/TMAP), which is optimized for aligning raw sequencing reads (from Ion Torrent) against the hg19 transcriptome reference sequence against regions defined in the Browser Extensible Display (BED) file (hg19_AmpliSeq_Transcriptome_21K_v1.bed). The quality of the raw data was evaluated based on three parameters: number of reads, mean read length and target detected (% of all amplicons that had ≥10 assigned reads). Differential gene expression analysis was performed using R/Bioconductor package edgeR with raw read counts from AmpliSeq. Read count normalization was performed using the counts per million (CPM) method. Significant differential expressed genes were called using the following threshold: absolute log fold-change ≥2 and Benjamini–Hochberg adjusted p < 0.05. The commercial software iPathway Guide (Advaita) was used for pathway analysis to explore significantly affected pathways.

**Immunocytochemistry**

An aliquot of harvested C-TACs were fixed on slides with 4% paraformaldehyde (pH 6.9, 20 min). Cell permeabilization was achieved with 0.3% Triton-X 100 (15 min), followed by blocking with 3% BSA (30 min). Cells were treated with primary antibodies (60 min), washed with PBS (pH 7.4), incubated with secondary antibodies (60 min), washed with PBS and then incubated with 4’,6-Diamidino-2-phenylindole dihydrochloride (DAPI) in dark (15 min). All incubations were at ambient temperature (20°C – 25°C). Positive and negative cell line controls were also processed with each batch of samples. ICC slides were scanned by Cell Insight CX7 High-Content Screening (HCS) Platform (Thermo Fisher Scientific, USA) which enables nuclear size filters and calibration of intensity thresholds for individual fluorophore conjugated antibodies. The intensity of each antigen expression was compared to batch controls (reference cell lines, Table 3). These precautions avoid or eliminate crosstalk in multiplexed analysis with different fluorophore conjugated antibodies. Aliquots of harvested cells were immunostained to determine status of EpCAM, Pan-CK and CD45 to confirm identity of C-TACs. Additional aliquots of harvested cells were then immunostained to determine status of theranostically relevant markers including VEGF, VEGFR, FGFR, PDGFR, EGFR, mTOR, AR, ER, HER2, TOPO and TUBB. Fluorescence imaging was performed on Cell Insight CX7 High-Content Screening Platform (ThermoFisher Scientific, USA).

**In vitro Chemoresponse Profiling**

The in vitro chemoresponse profiling (CRP) assay was designed^2^ to evaluate the sensitivity of viable TDCs and CTACs to various anticancer agents (ACA). The test concentration for each ACA was based on reported peak plasma concentration at the recommended clinical dose and was preliminarily evaluated on SKBR3 (ATCC® HTB-30™), SW620 (ATCC® CCL-227™) and RCC 769-P (ATCC® CRL-1933™) cell lines and has been reported previously^2^.

**TDCs:** Approximately 10^4^ cells/well were seeded into 96-well culture plates and treated with ACAs at 37^°^C, for 30 min, under 5% CO_2_ and 4% O_2_. The plates were transferred into the incubator chamber of a microplate reader (VarioScan LUX, Thermo Fisher Scientific) where absorbance (λ = 600 nm) was recorded every 5 min over 12 h. Change in absorbance which correlates with apoptosis was converted to kinetic units (KU) of apoptosis as described previously^3^. Baseline apoptotic events were accounted for by using control wells with untreated cells. Active apoptosis was indicated as > 1.0 KU.

**C-TACs:** Approximately 100 C-TACs/well were seeded into 96 well culture plates and incubated for 24 hours (37^°^C, 5% CO_2_, 4% O_2_). Viable cells were stained with Calcein AM and treated with ACAs. The plates were placed in the on-stage incubator of fluorescent microscope EVOS M7000 (Thermo Fisher Scientific) at 37^°^C with 5% CO_2_, 4% O_2_ and the wells imaged every 10 min for 12 h Extent of cell death was determined based on cell morphology changes and time required for fade out of live cell tracking dye.

All in vitro CRP assay plates included control wells (no drug) to determine baseline mortality as well as positive (known cytotoxicity) controls with SKBR3, SW620 or RCC 769-P cells. Cell death above 50% or >5KU after subtraction of baseline mortality in control wells were considered as an indication of drug efficacy. Drugs with higher activity were shortlisted for incorporation into patient regimens.

**References**

1. Akolkar D, Patil D, Crook T, et al. Circulating ensembles of tumor-associated cells: A redoubtable new systemic hallmark of cancer. International Journal of Cancer. 2020 Jun;146(12):3485-3494.
2. Crook, T., Gaya, A., Page, R. et al. Clinical utility of circulating tumor-associated cells to predict and monitor chemo-response in solid tumors. Cancer Chemother Pharmacol (2020). https://doi.org/10.1007/s00280-020-04189-8
3. Kravtsov V, Fabian I. Automated monitoring of apoptosis in suspension cell cultures. Lab Invest. 1996 Feb;74(2):557-70.
